# Activity of Angiogenesis Inhibitors in Metastatic Epithelioid Hemangioendothelioma: A Case Report

**DOI:** 10.3969/j.issn.2095-3941.2012.02.010

**Published:** 2012-06

**Authors:** Sumit Gaur, Alireza Torabi, Thomas J O’Neill

**Affiliations:** 1 Divisions of Hematology-Oncology, Texas Tech University Health Science Center, TX 79905, USA; 2 Divisions of Pathology, Texas Tech University Health Science Center, TX 79905, USA; 3 Divisions of Radiology, Texas Tech University Health Science Center, TX 79905, USA

**Keywords:** hemangioendothelioma, angiogenesis inhibitors, nab-paclitaxel, bevacizumab

## Abstract

This report describes a patient with metastatic epithelioid hemangioendothelioma treated with bevacizumab and nanoparticle albumin-bound paclitaxel. The treatment was well tolerated and led to the stabilization of an aggressive variant of the disease. This case report is the first one that describes the activity of the combination of chemotherapy and bevacizumab in epithelioid hemangioendothelioma. Literature describing the activity of bevacizumab and other agents (thalidomide, lenalidomide, and interferon) believed to possess anti-angiogenic activities is also reviewed.

## Introduction

Epithelioid hemangioendothelioma (EHE) is a rare vascular tumor with an incidence of less than one in a million. Considering the rarity of this disease, there is no published large trial to guide therapy. Surgical resection is generally performed for localized disease. The management of metastatic disease is difficult because it is generally resistant to most chemotherapeutic agents. Histologically, the tumor shows proliferation of epithelioid cells, which expresses vascular markers such as CD31, CD34, and Factor VIII. Consequently, interest in employing anti-angiogenic therapy for this disease is increasing. Bevacizumab, a humanized monoclonal antibody directed against vascular endothelial growth factor, shows activity in numerous solid malignancies. This case report describes a patient with metastatic thoracic EHE who was treated with bevacizumab and nanoparticle albumin-bound (nab) paclitaxel. Literature describing the activity of bevacizumab and other anti-angiogenic agents in EHE is also reviewed.

## Case Report

**Figure 1:**
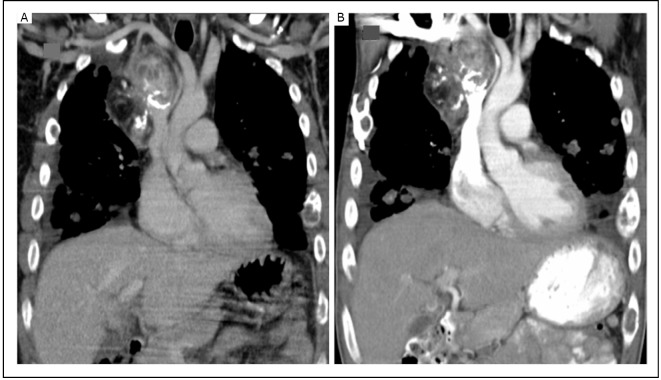
A: CT scan (coronal view) showing a mass in the anterior and superior mediastinum containing a solid-enhancing component, coarse calcification, and fat. The mass involves the superior vena cava and right brachiocephalic vein. B: CT scan after 6 months showing stable appearance.

**Figure 2:**
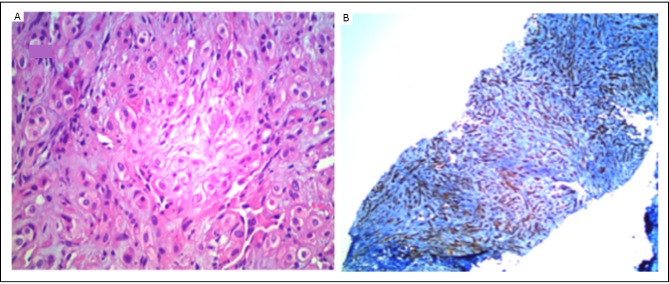
A: Nests of rounded and slightly spindled epithelioid cells embedded in a myxoid matrix H&E (× 400). B: CD34 immunostaining (× 100). The tumor cells stained for CD34 are consistent with a vascular origin.

A 35-year old Hispanic male presented with a 12-month history of cough, shortness of breath, and pain involving the chest wall, lower back, and both legs. He lost 10 kg of his body weight over this time period. He did not smoke and had no other significant medical history. Physical examination was remarkable for 88% oxygen saturation in room air. He had crackles in both lung fields. Computed tomography (CT) of the thorax (**Figure 1A**) showed a heterogeneous mass in the superior and anterior mediastinum that involved the superior vena cava and right brachiocephalic vein. Innumerable 1-2 cm pulmonary nodules were present in both lung fields. A bone scan showed extensive metastatic disease in the calvarium, sternum, ribs, pelvis, spine, and femur. Magnetic resonance imaging of the head did not show brain involvement. CT-guided core biopsy of the mediastinal mass showed nests of rounded and slightly spindled epithelioid cells embedded in a myxoid matrix. Immunostains were positive for CD31 and CD34, consistent with EHE (**Figure 2**). The alpha-fetoprotein and beta-human chorionic gonadotropin levels were normal.

He received palliative radiotherapy on the sites of painful metastases in his legs and pelvis. Subsequently, he was treated with nab-paclitaxel at a dose of 100 mg/m^2^ on a weekly schedule and bevacizumab at a dose of 15 mg/kg every 3 weeks. In view of extensive bony metastases, he also received zoledronic acid infusions. Treatment was well tolerated and led to improvement in his pain and exercise tolerance. Interim CT scans after 3 and 6 months of therapy indicated stable disease with no new metastatic lesion (**Figure 1B**). Nab-paclitaxel was discontinued after 6 months to avoid cumulative neurotoxicity, but bevacizumab was continued.

## Discussion

The World Health Organization categorizes EHE as a malignant vascular neoplasm ^[^^1^^]^. However, the clinical spectrum of this disease is variable and a more accepted concept is that its malignant potential varies between those of an indolent hemangioma and an aggressive angiosarcoma^[^^2^^]^. Pro-angiogenic factors are believed to promote the growth of these vascular tumors ^[^^3^^, ^^4^^]^. Given that chemotherapy is generally ineffective in EHE, angiogenesis inhibition is a reasonable approach to manage patients with metastatic EHE.

A PubMed search using the keywords “hemangioen-dothelioma” and “bevacizumab” identified a total of 6 case reports (**Table 1**). Five cases involved patients with thoracic primaries ^[^^5^^-^^8^^]^, and one patient had a skeletal EHE^[^^9^^]^. One had partial response for 13 months, 1 had stable disease, and 4 had progressive disease. Bevacizumab was believed to cause cerebral infarction and death in one patient ^[^^7^^]^. Carboplatin and paclitaxel were the most commonly used chemotherapeutic agents with bevacizumab. We treated our patient with both nab-paclitaxel and bevacizumab. This formulation of paclitaxel has many theoretical advantages over the solvent-based formulation. It enters the tumor endothelium by glycoprotein 60-caveolin-1-mediated endocytosis. This targeting of the endothelium can lead to anti-angiogenic activity that can be further potentiated by bevacizumab ^[^^10^^]^. The leaky endothelial junctions of the tumor allow for greater permeation of nab-paclitaxel than solvent mixed paclitaxel ^[^^11^^]^. The drug can also be retained longer in the tumor by binding to secreted protein acid rich cysteine, which is present in tumor stroma ^[^^12^^]^. The treatment was well tolerated and led to disease stabilization without significant toxicity.

**Table 1:** Bevacizumab in epithelioid hemangioendothelioma.

Author	Reference	Age (years)	Gender (M/F)	Dose and schedule	Chemotherapy	Outcome	Follow-up
Belmont et al.	5	41	M	15 mg/kg, every 21 days	Carboplatin, paclitaxel, docetaxel	Partial response	13 months
Kim YH et al.	6	44	F	15 mg/kg, every 21 days	Carboplatin,paclitaxel	Progression	
Mizota et al.	7	59	F	15 mg/kg, every 21 days	Carboplatin, paclitaxel	Progression	3 months
Lazarus et al.	8	42	M	?	Paclitaxel	Progression	1-2 months
Lazarus et al.	8	42	M	?	Carboplatin, etoposide	Progression	2-3 months
Trautmann et al.	9	19	F	7.5 mg/m^2^, every 21 days	None	Stable disease	16 months

Although the mechanism of action of thalidomide and its analog, lenalidomide, are not fully understood, they are believed to have immunomodulatory and anti-angiogenic properties. A PubMed search using “thalidomide” and “hemangioenothelioma” identified 5 case reports, whereas “lenalidomide” and “hemangioendothelioma” resulted in 1 case report (**Table 2**). Among them, 6 had hepatic primary and 1 had multifocal skin and central nervous system involvement ^[^^13^^-^^18^^]^. None had thoracic primary involvement. Two cases had partial responses, which lasted up to 9 years in 1 case. Another two patients had stable disease lasting up to 7 years. The dose of thalidomide varied from 300 mg daily to 400 mg twice a day. At these doses, thalidomide can induce prohibitive toxicity and cannot be recommended for all patients. Lenalidomide is a better tolerated alternative that warrants further exploration. The use of immunomodulation in pulmonary EHE is also worthy of further study.

**Table 2:** Thalidomide and lenalidomide (IMID’s) in epithelioid hemangioendothelioma.

Author	Reference	Age (years)	Gender (M/F)	IMID	Dose and schedule	Outcome	Follow-up
Salech F el al.	13	40	F	Thalidomide	300mg daily	Partial response	109 months
Raphael el al.	14	53	F	Thalidomide	400 mg daily	Stable disease	84 months
Kassam el al.	15	13	F	Thalidomide	400 mg twice a day	Progressive disease	_
Bolke et al.	16	47	M	Thalidomide	?	Progressive disease/death	--
Mascarenhas el al.	17	52	M	Thalidomide	?	Partial response	--
Sumrall et al.	18	31	F	Lenalidomide	25 mg daily for 21/28 days	Stable disease	48 months

Interferon alpha, which has anti-angiogenic properties, is also used in EHE. **Table 3** summarizes the literature on the use of interferon over the last 10 years ^[^^19^^-^^25^^]^. We excluded case reports describing adjuvant use of interferon after surgical resection because the efficacy of the drug in these case reports cannot be evaluated. Remarkably, 1 patient with EHE of breast metastasis to the lungs had a complete clinical response to interferon. She was treated with bilateral mastectomies and subsequently given interferon. The authors described a complete clinical response that lasted for 7 years^[^^23^^]^. Reduced metastatic lesion sizes after primary tumor removal has been described in multiple malignancies. In patients with metastatic EHE who have an easily resectable primary tumor, this approach may be reasonable.

**Table 3:** Interferon in epithelioid hemangioendothelioma.

Author	Reference	Primary site	Interferon	Dose and schedule	Outcome	Follow-up
Radzikowaska et al.	19	Thoracic	Alpha-2a	3 million units, 3 times a week	Stable disease	3 months
Saleiro et al.	20	Thoracic	Alpha 2b	-	Progressive disease	9 months
Calabro et al.	21	Thoracic	Alpha 2a	-	Stable disease	-
Hassan et al.	22	Thyroid	Alpha	3 million units, 5 times a week	Progressive disease	2 months
Marsh et al.	23	Breast	Alpha	3 millon units, 5 days a week for 1 year	Complete response	84 months
Al-Sharim et al.	24	Thoracic	Alpha	7 million units, 3 times a week.	Progressive disease	2 months
Kayler et al.	25	Hepatic	Alpha-2b	3 million units daily	Partial response	4 months

A better understanding of the pathogenesis of these neoplasms and large clinical trials are needed to develop enhanced therapeutic approaches. Recently, chromosomal translocation involving t(1;3)(p36;q25) leading to a fusion gene WWTR1/CAMTA1 was described in virtually all patients with EHE ^[^^26^^]^. Nevertheless, anti-angiogenesis therapy presently remains a valid approach for managing EHE.
